# Basal and one-month differed neutrophil, lymphocyte and platelet values and their ratios strongly predict the efficacy of checkpoint inhibitors immunotherapy in patients with advanced BRAF wild-type melanoma

**DOI:** 10.1186/s12967-022-03359-x

**Published:** 2022-04-05

**Authors:** Michele Guida, Nicola Bartolomeo, Davide Quaresmini, Pietro Quaglino, Gabriele Madonna, Jacopo Pigozzo, Anna Maria Di Giacomo, Alessandro Marco Minisini, Marco Tucci, Francesco Spagnolo, Marcella Occelli, Laura Ridolfi, Paola Queirolo, Ivana De Risi, Monica Valente, Angela Monica Sciacovelli, Vanna Chiarion Sileni, Paolo Antonio Ascierto, Lucia Stigliano, Sabino Strippoli

**Affiliations:** 1 Rare Tumors and Melanoma Unit, IRCCS Istituto Tumori “Giovanni Paolo II”, Viale O. Flacco, 6570124 Bari, Italy; 2 Department of Biomedical Sciences and Human Oncology, University of Bari, Bari, Italy; 3 Department of Medical Sciences, Dermatologic Clinic, University of Turin, Turin, Italy; 4 Department of Melanoma, Cancer Immunotherapy and Development Therapeutics, Istituto Nazionale Tumori IRCCS Fondazione “G. Pascale”, Napoli, Italy; 5 Melanoma Oncology Unit, Veneto Institute of Oncology IOV-IRCCS, Padova, Italy; 6 Center for Immuno-Oncology, University Hospital of Siena, University of Siena, Siena, Italy; 7 Department of Oncology, Azienda Sanitaria Universitaria del Friuli Centrale, Udine, Italy; 8 Medical Oncology Unit, University of Bari Aldo Moro, Bari, Italy; 9 Skin Cancer Unit, IRCCS Ospedale Policlinico San Martino, Genova, Italy; 10 Oncology Unit, Azienda Ospedaliera Santa Croce e Carle, Cuneo, Italy; 11 Immunotherapy, Cell Therapy and Biobank Unit, IRCCS Istituto Romagnolo Per Lo Studio Dei Tumori (IRST) “Dino Amadori”, Meldola, Italy; 12 Division of Melanoma Sarcoma and Rare Tumors, IEO European Institute of Oncology IRCCS Milan, Milan, Italy; 13 Center for Immuno-Oncology, Medical Oncology and Immunotherapy, Department of Oncology, University Hospital of Siena, Siena, Italy; 14 Rare Tumors and Melanoma Unit, IRCCS Istituto Tumori “Giovanni Paolo II”, Bari, Italy; 15 IRCCS Istituto Tumori “Giovanni Paolo II”, Bari, Italy

**Keywords:** Neutrophil-to-lymphocyte ratio, Platelet-to-lymphocyte ratio, Metastatic melanoma, Checkpoint inhibitors

## Abstract

**Background:**

To evaluate the capability of basal and one-month differed white blood cells (WBC), neutrophil, lymphocyte and platelet values and their ratios (neutrophils-to-lymphocytes ratio, NLR, and platelets-to-lymphocytes ratio, PLR) in predicting the response to **i**mmune checkpoint inhibitors (ICI) in metastatic melanoma (MM).

**Methods:**

We performed a retrospective study of 272 BRAF wild-type MM patients treated with first line ICI. Bivariable analysis was used to correlate patient/tumor characteristics with clinical outcomes. Variations between time 1 and time 0 (Δ) of blood parameters were also calculated and dichotomized using cut-off values assessed by ROC curve.

**Results:**

At baseline, higher neutrophils and NLR negatively correlated with PFS, OS and disease control rate (DCR). Higher PLR was also associated with worse OS. In multivariable analysis, neutrophils (p = 0.003), WBC (p = 0.069) and LDH (p = 0.07) maintained their impact on PFS, while OS was affected by LDH (p < 0.001), neutrophils (p < 0.001) and PLR (p = 0.022), while DCR by LDH (p = 0.03) and neutrophils (p = 0.004). In the longitudinal analysis, PFS negatively correlated with higher Δplatelets (p = 0.039), ΔWBC (p < 0.001), and Δneutrophils (p = 0.020), and with lower Δlymphocytes (p < 0.001). Moreover, higher ΔNLR and ΔPLR identified patients with worse PFS, OS and DCR. In the multivariable model, only ΔNLR influenced PFS (p = 0.004), while OS resulted affected by higher ΔWBC (p < 0.001) and lower Δlymphocytes (p = 0.038). Higher ΔWBC also affected the DCR (p = 0.003). When clustering patients in 4 categories using basal LDH and ΔNLR, normal LDH/lower ΔNLR showed a higher PFS than high LDH/higher ΔNLR (20 vs 5 months). Moreover, normal LDH/higher Δlymphocytes had a higher OS than high LDH/lower Δlymphocytes (50 vs. 10 months).

**Conclusions:**

Baseline and early variations of blood cells, together with basal LDH, strongly predict the efficacy of ICI in MM. Our findings propose simple, inexpensive biomarkers for a better selection of patient treatments. Prospective multicenter studies are warranted to confirm these data.

## Background

The medical treatment of metastatic melanoma (MM) has significantly improved thanks to the identification of specific genetic alterations which became important therapeutic targets for new treatments as the combination of anti-BRAF + anti-MEK drugs for patients harbouring BRAF V600 mutations [1], and thanks to the new immunological approaches with monoclonal antibodies directed against the immune checkpoint molecules CTLA-4 (cytotoxic T lymphocyte antigen-4) and PD-1 (programmed death antigen 1) or its ligand PD-L-1. The anti-CTLA-4 ipilimumab is able to induce a response rate of approximately 15% with about 20% of patients remaining long-term responders [2]. The anti-PD-1 nivolumab and pembrolizumab, alone or in combination with ipilimumab, provide higher response rates of approximately 40% and almost 60%, respectively, with the majority of responses being durable [3, 4]. Despite these results, more than a half of patients do not respond or relapse early to checkpoint inhibitors and their prognosis is very poor.

Several potential biomarkers have been studied in the effort to select responder patients from non-responders with the aim to avoid useless high-cost therapies and potentially severe adverse events in non-responders. Unfortunately, no validated biomarkers are currently available in melanoma.

Serum lactate dehydrogenase (LDH) is the first prognostic blood biomarker included in the American Joint Committee on Cancer (AJCC) staging system for patients with MM. LDH is an essential enzyme for melanoma cells metabolism catalyzing the reversible conversion of pyruvate into lactate. Also, the accumulated lactate was proved to promote tumor immune escape by reducing the survival and cytolytic capacity of CD8 + T cells and natural killer cells. LDH resulted a strong prognostic marker independent of type of treatment. The prognostic value of LDH was confirmed in several pivotal and real-life studies using ICI (1–4).

Among more recent biomarkers, only higher levels of PD-L-1 expression seems correlated with a better response, but their impact on overall survival is still questionable [5].

In recent years, there has been an increasing interest in establishing the role of basal white blood cells (WBC), neutrophil, lymphocyte and platelet count and the neutrophil-to-lymphocyte ratio (NLR) and platelet-to-lymphocyte ratio (PLR) as prognostic and predictive biomarkers to immune checkpoint inhibitors (ICI). These findings derive from the known contribution of neutrophils and platelets to cancer cell growth and migration, and from the evidence of the inter-relationships between thrombosis, inflammation and cancer immune surveillance [6–11]. Beyond their crucial role in hemostasis and thrombosis, platelets are increasingly recognized as regulators of inflammation, angiogenesis and immunomodulation in cancer progression. Furthermore, platelets cover the cancer cells, inducing their protection from the attack of the immune cells. In this way, in addition to enhancing the metastatic power of the tumor, they increase the risk of formation of microthrombi and embolic events. Through the modulation of the immune system and the interaction with leukocytes, platelets influence inflammatory processes in cancer at various stages: by altering the activation state of the endothelium, recruiting leukocytes in tumor sites and tuning the inflammatory environment in sites of primary and metastatic tumors. Finally, platelets modulate innate leukocyte effector functions such as antigen presentation by dendritic cells, monocyte recruitment and differentiation or neutrophil extracellular trap formation. Finally, platelets and their microvesicles have further been shown to inhibit activation of T-helper cell and natural killer cell functions [8–11].

High baseline levels of NLR and PLR alone or combined have been associated with poor survival and high risk of recurrence regardless of cancer type [12, 13]. In addition, they appear to predict worse efficacy both of chemotherapy and immunotherapy suggesting their potential role as agnostic markers of efficacy [14–16].

Regarding melanoma, the prognostic role of pre-treatment NLR on PFS and OS has been reported in a large meta-analysis of patients at different stages of disease, showing a negative role independently of the disease stage and the type of treatments used [17]. The prognostic value of pre-treatment NLR in the era of ICI therapy has been evaluated in small subsets of patients affected by different type of neoplasms. However, the utility of NLR in the context of the modern immunotherapy has not been well-defined. In metastatic disease higher level of basal neutrophil and NLR resulted associated with poorer clinical outcomes in patients receiving ipilimumab [18–20] or anti-PD-1 treatment [21–23].

The prognostic and predictive role of platelets in melanoma has been less investigated and the modest data published so far are controversial [15, 24, 25].

Finally, it is still debated if early changes in hematological parameters during ICI therapy could increase the magnitude in discriminating responder from non-responder patients. In this regard, very few and conflicting data are available yet [15, 16, 25–27].

Our study aimed to evaluate neutrophil, lymphocyte and platelet values and their ratios both at baseline and after one course of ICI therapy in order to verify if their variations could increase their prognostic and predictive value in patients with MM treated with ICI.

Patients with the BRAF mutation were excluded from our analysis because most of them were treated as first line with anti-BRAF/antiMEK targeted therapy. We thought that this pre-treatment would have confounding effects on clinical outcomes leading to a strong bias of patient selection (only progressing patients would have been included in the study analysis).

## Patients and methods

### Patients and study design

We retrospectively recruited 272 patients from 11 referral Centers in Italy. After the approval by its Ethical Committee, each Center recorded the clinical data of its patients in an anonymized electronic database. All data were then collected in a central database at the IRCCS Istituto Tumori “Giovanni Paolo II” of Bari, Italy, and submitted to the statistical analysis.

The main inclusion criteria were diagnosis of metastatic melanoma, first-line treatment with checkpoint inhibitors and presence of measurable disease according to RECIST 1.1 criteria [28]. ICI therapy included the anti-PD-1 nivolumab and pembrolizumab or the anti-CTLA-4 antibody ipilimumab, or the combination of nivolumab plus ipilimumab (Table 1).

**Table 1:** Baseline characteristics of patients (n = 272)

Characteristic	n (%)
Age at diagnosis, years, median [IQR]	63.2 [52.0;73.0]
Age at metastasis, years, median [IQR]	67.0 [55.0;75.0]
Male sex, n (%)	172 (63.2)
Basal LDH, n (%)	
Normal	171 (62.87)
Increased	79 (29)
Not available	22 (8.1)
Mutational status	
BRAF/NRAS wild type	145 (53.3)
NRAS mutated	127 (46.7)
N of metastatic sites < 3, n (%)	169 (62.1)
Site of melanoma	
Cutaneous	210 (77.2)
Mucosal	19 (7)
Ocular	14 (5.1)
Unknown	29 (10.7)
Prior adjuvant therapy	20 (7.3%)
ECOG PS	
0	192 (70.6)
1	78 (28.7)
2	2 (0.7)
Stage at metastatic disease (AJCC VIII edition)	
M1a	66 (24.3)
M1b	77 (28.3)
M1c	102 (37.5)
M1d	27 (9.9)
First line therapy	
Anti-PD-1	209 (76.8)
Anti-CTLA-4	57 (20.9)
Anti-PD-1 + Anti-CTLA-4	6 (2.2)

*IQR* interquartile range

All drugs were administered intravenously according to the standard doses and schedules from January 2011 to August 2019.

Patients with BRAF mutated melanomas were excluded to avoid the possible confounding impact of anti-BRAF/anti-MEK-targeted therapy on clinical outcomes. In fact, treatment with targeted therapy is normally used as first line therapy in patients harboring BRAF V600 mutation.

The main recorded patient characteristics included sex, age at diagnosis, site and characteristics of primary cancer, prior systemic adjuvant therapy, BRAF/NRAS genotype, age at metastatic disease, Eastern Cooperative Oncology Group (ECOG) performance status at the beginning of therapy, metastatic stage and sites of metastases, presence of brain metastases, lactate dehydrogenase (LDH) level at metastatic disease, and subsequent therapies after first-line treatment.

Complete blood cell count was obtained at the start of treatment and before the subsequent cycle of treatment. LDH values were only available at baseline, and were considered normal if inferior to the upper limit of normal range (< ULN) or elevated if superior to the upper limit of normal (> ULN).

We defined baseline neutrophil to lymphocyte ratio (NLR) and platelets to lymphocyte ratio (PLR) as neutrophil count divided by lymphocyte count and platelet count divided by lymphocyte count, respectively. The cut-off values used in our analyses were derived from the ROC curves of the overall survival. We also collected cell blood counts after the first dose of immunotherapy (21 days for pembrolizumab and ipilimumab or 28 days for nivolumab) and evaluated their relative changes (Δ) with clinical outcomes to capture eventual modifications induced by a brief course of checkpoint inhibitors therapy.

We correlated the hematological data with the patients’ characteristics, tumor features and clinical outcomes including overall survival (OS), progression-free survival (PFS), overall response rate (ORR) and disease control rate (DCR). Objective tumor responses were performed every 3 months and were assessed by investigators using computed tomography or magnetic resonance imaging as per Response Evaluation Criteria in Solid Tumors version 1.1 (RECIST v1.1) [28] and reported as complete response (CR), partial response (PR), stable disease (SD), and progressive disease (PD). We evaluated the overall response rate (ORR) as CR plus PR, and the disease control rate (DCR) as CR plus PR plus SD disease lasting more than 6 months. Patients with SD inferior to 6 months were included in the PD group. DFI, PFS and OS were also evaluated in all patients.

### Statistical analysis

PFS was defined as the time from the first cycle of immune therapy to progression or death. OS was calculated from the date of the first immune therapy administration to the date of death for any reason. Patients alive at the last date of follow-up were censored for the final OS analysis. Similarly, patients alive and progression-free were censored for the final PFS analysis.

The Shapiro–Wilk test was used to determine whether the continuous parameters (peripheral white blood count, neutrophil count, platelet count, lymphocyte count, neutrophils/lymphocytes (NLR) and platelets/lymphocytes ratios (PLR)) showed a normal distribution and consequently they were expressed as the median and interquartile range (IQR). Non-parametric Mann–Whitney U Test was used to compare the values of these parameters at baseline and after one-month, then the differences between the values at baseline and those after one month were dichotomized. In order to define common and constant cut-off values for all parameters, (ROC) curves were performed using the OS as a classification criterion, then the resulting cut-off values were applied to PFS and DCR.

Through a two-variable logistic regression model, we tested the effect of each variable at baseline and its dichotomized difference at one month on the probability of a positive DCR. The effect of each parameter at baseline and its dichotomized difference at 3–4 weeks on PFS and OS was assessed using the two-variable Cox-regression model, and the proportional hazard assumptions for the Cox model were checked. All variables together with age at diagnosis, sex, number of sites and LDH were included in multivariable logistic regression model to evaluate the DCR and in multivariable Cox-regression model to evaluate PFS and OS outcomes. Stepwise selection using Akaike Information Criterion (AIC) was used to estimate the final models. The results of the logistic model and the Cox model were expressed respectively by the Odds Ratios (OR) and the Hazard Ratios (HR), their 95% Wald confidence intervals, and the p-values of the Wald chi-square tests.

All tests of statistical significance were two-tailed, and p-values < 0.05 were considered statistically significant. Statistical analysis was performed using SAS 9.4 software [29].

## Results

### Treatments and clinical outcomes

Of all 272 patients, 209 (76.8%) were treated with anti-PD-1, while 57 patients (20.9%) with anti-CTLA-4 and only 6 patients (2.2%) with the combination of anti-PDL-1 plus anti-CTLA-4. The median age of patients was 67 years [IQR 55–75] with the 63.2% being men. The primary melanoma site was skin in 77.2%, mucosae in 7%, choroid in 5.1%, unknown in 10.7%. A large majority (92.7%) of patients did not undergo adjuvant therapy and had an ECOG performance status of 0 or 1 (99%). Regarding the metastatic stage, M1c disease was the most common (37.5%), while 9.9% of patients presented brain metastases (Table 1).

The response to therapy included 114 major responses (41.9%) with a disease control in 154 patients (56.6%). The median PFS was 10 [IQR; 6–15] months and the OS was 29 [IQR; 22–43] months (Table 2). When we recalculated PFS and OS by excluding patients with worse prognosis (brain metastases, mucosal melanoma, uveal melanoma), we reported a median PFS of 12 [7–19] months and a median OS of 42 [27–61] months.

**Table 2:** Clinical outcomes of the entire population to checkpoint inhibitors immunotherapy

Clinical outcomes	Patients, n (%)
ORR	114 (41.9)
SD	40 (14.7)
DCR	154 (56.6)
PD	118 (43.4)
PFS, median [IQR]	10 [6–15]
OS, median [IQR]	29 [22–43]

*ORR* overall response rate, *SD* stable disease lasting 6 months or more, *DCR* disease control rate (major responses + SD lasting 6 months or more), *PD* progressive disease (non-responders + SD lasting less than 6 months), *PFS* progression free survival, *OS* overall survival

### Correlation between baseline parameters and clinical outcomes

We first investigated the possible correlations between baseline characteristics of patients and disease such as age, sex, number of metastatic sites (< 3 vs ≥ 3), LDH (normal *vs* elevated), and clinical outcomes.

In the univariate analysis, a longer PFS was associated with a lower number of metastatic sites and lower LDH levels (p = 0.013 and p = 0.001, respectively) (Table 3).

**Table 3:** Effect of each parameter on PFS according to baseline values and to the variation between T1 value (after 1 month of therapy) and T0 value (baseline value). This difference has been indicated as delta value (Δ)

Parameter			Univariate				Two-variable				Multivariable^a^			
Variable	Timepoint	Value	p	HR	Lower HR	Upper HR	p	HR	Lower HR	Upper HR	p	HR	Lower HR	Upper HR
Platelets	Basal	+ 10,000					0.419	1.007	0.900	1.025	–	–	–	–
	Δ	Ref ≤ 25,000					**0.039**	**1.398**	**1.016**	**1.922**	0.116	1.310	0.935	1.836
White blood cell count	Basal	+ 100					**0.001**	**1.006**	**1.002**	**1.010**	0.069	0.990	0.979	1.001
	Δ	Ref ≤ 1920					** < 0.001**	**2.063**	**1.396**	**3.049**	–	–	–	–
Neutrophils	Basal	+ 100					** < 0.001**	**1.011**	**1.007**	**1.015**	**0.003**	**1.022**	**1.008**	**1.037**
	Δ	Ref ≤ 310					**0.020**	**1.429**	**1.058**	**1.930**	–	–	–	–
Lymphocytes	Basal	+ 100					0.054	0.981	0.962	1.000	–	–	–	–
	Δ	Ref ≥ -300					** < 0.001**	**1.910**	**1.356**	**2.692**	–	–	–	–
NL ratio	Basal	+ 1					** < 0.001**	**1.176**	**1.078**	**1.284**	–	–	–	–
	Δ	Ref ≤ 0.86					** < 0.001**	**1.975**	**1.411**	**2.764**	**0.004**	**1.709**	**1.183**	**2.468**
PL ratio	Basal	+ 1					0.156	1.001	0.999	1.003	–	–	–	–
	Δ	Ref ≤ 22.85					**0.001**	**1.693**	**1.240**	**2.311**	–	–	–	–
Age	Basal	+ 1	0.210	1.007	0.996	1.019					–	–	–	–
Sex	Basal	Ref F	0.601	0.923	0.682	1.248					–	–	–	–
Number of metastatic sites	Basal	Ref ≥ 3	**0.013**	**0.686**	**0.509**	**0.925**					–	–	–	–
LDH	Basal	Ref elevated	**0.001**	**0.581**	**0.420**	**0.803**					0.070	0.729	0.518	1.026

Statistically significant values in bold

At the Δ timepoint the variables have been dichotomized and the “value” represents the reference category

HR (hazard ratio) values are expressed in terms of risk of progression

^a^After stepwise selection

Effect of each parameter on PFS according to baseline values and to the variation between T1 value (after 1 month of therapy) and T0 value (baseline value). This difference has been indicated as delta value (Δ).

The OS was also positively affected by lower LDH levels (p < 0.001), while the number of metastatic sites had no statistical significance (p = 0.089) (Table 4).

**Table 4:** Effect of each parameter on OS according to baseline values and to the variation between T1 value (after 1 month of therapy) and T0 value (baseline value). This difference has been indicated as delta value (Δ)

Parameter			Univariate				Two-variable				Multivariable^a^			
Variable	Timepoint	Value	p	HR	Lower HR	Upper HR	p	HR	Lower HR	Upper HR	p	HR	Lower HR	Upper HR
Platelets	Basal	+ 10,000					**0.041**	**1.020**	**1.001**	**1.039**	–	–	–	–
	Δ	Ref ≤ 25,000					**0.022**	**1.512**	**1.063**	**2.151**	–	–	–	–
White blood cell count	Basal	+ 100					** < 0.001**	**1.009**	**1.005**	**1.013**	–	–	–	–
	Δ	Ref ≤ 1920					** < 0.001**	**3.246**	**2.171**	**4.854**	** < 0.001**	**2.918**	**1.882**	**4.526**
Neutrophils	Basal	+ 100					** < 0.001**	**1.014**	**1.010**	**1.018**	** < 0.001**	**1.011**	**1.005**	**1.016**
	Δ	Ref ≤ 310					** < 0.001**	**1.880**	**1.330**	**2.659**	–	–	–	–
Lymphocytes	Basal	+ 100					**0.045**	**0.977**	**0.955**	**0.999**	–	–	–	–
	Δ	Ref ≥ -300					** < 0.001**	**2.319**	**1.578**	**3.409**	**0.038**	**1.627**	**1.028**	**2.576**
NL ratio	Basal	+ 1					** < 0.001**	**1.279**	**1.169**	**1.400**	–	–	–	–
	Δ	Ref ≤ 0.86					** < 0.001**	**2.908**	**2.012**	**4.202**	–	–	–	–
PL ratio	Basal	+ 1					**0.006**	**1.003**	**1.001**	**1.005**	**0.022**	**1.003**	**1.000**	**1.005**
	Δ	Ref ≤ 22.85					** < 0.001**	**2.019**	**1.429**	**2.853**	0.140	1.378	0.901	2.109
Age	Basal	+ 1	0.255	1.008	0.994	1.021					–	–	–	–
Sex	Basal	Ref F	0.282	0.829	0.589	1.166					–	–	–	–
Number of metastatic sites	Basal	Ref ≥ 3	0.089	0.746	0.532	1.046					–	–	–	–
LDH	Basal	Ref elevated	** < 0.001**	**0.395**	**0.276**	**0.564**					** < 0.001**	**0.499**	**0.344**	**0.724**

Statistically significant values in bold

At the Δ timepoint the variables have been dichotomized and the “value” represents the reference category

HR (hazard ratio) values are expressed in terms of risk of mortality

^a^After stepwise selection

The response to therapy resulted also inversely influenced by the number of metastatic sites (p = 0.010) and LDH levels (p = 0.002**)** (Table 5).

**Table 5:** Effect of each parameter on DCR according to baseline values and to the variation between T1 value (after 1 month of therapy) and T0 value (baseline value). This difference has been indicated as delta value (Δ)

Parameter			Univariate				Two-variable				Multivariable^a^			
Variable	Timepoint	Value	p	OR	Lower OR	Upper OR	p	OR	Lower OR	Upper OR	p	OR	Lower OR	Upper OR
Platelets	Basal	+ 10,000					0.077	1.027	0.997	1.058	–	–	–	–
	Δ	Ref ≤ 25,000					**0.043**	**1.749**	**1.019**	**3.001**	–	–	–	–
White blood cell count	Basal	+ 100					**0.002**	**1.017**	**1.006**	**1.028**	–	–	–	–
	Δ	Ref ≤ 1920					** < 0.001**	**4.136**	**1.927**	**8.878**	**0.003**	**3.422**	**1.528**	**7.666**
Neutrophils	Basal	+ 100					** < 0.001**	**1.030**	**1.015**	**1.044**	**0.004**	**1.022**	**1.007**	**1.037**
	Δ	Ref ≤ 310					**0.021**	**1.850**	**1.097**	**3.120**	–	–	–	–
Lymphocytes	Basal	+ 100					0.381	0.987	0.957	1.017	–	–	–	–
	Δ	Ref ≥ − 300					**0.002**	**2.621**	**1.444**	**4.756**	0.074	1.804	0.944	**3.446**
NL ratio	Basal	+ 1					** < 0.001**	**1.329**	**1.127**	**1.568**	–	–	–	–
	Δ	Ref ≤ 0.86					**0.001**	**2.779**	**1.503**	**5.140**	–	–	–	–
PL ratio	Basal	+ 1					0.086	1.003	1.000	1.006	–	–	–	–
	Δ	Ref ≤ 22.85					**0.009**	**2.022**	**1.191**	**3.432**	–	–	–	–
Age	Basal	+ 1	0.879	1.001	0.983	1.020					–	–	–	–
Sex	Basal	Ref F	0.507	0.845	0.514	1.389					–	–	–	–
Number of metastatic sites	Basal	Ref ≥ 3	**0.010**	**0.519**	**0.316**	**0.853**					–	–	–	–
LDH	Basal	Ref elevated	**0.002**	**0.419**	**0.243**	**0.722**					**0.032**	**0.528**	**0.295**	**0.946**

Statistically significant values in bold

At the Δ timepoint the variables have been dichotomized and the “value” represents the reference category

OR (odds ratio) values are expressed in terms of risk of non-response

^a^After stepwise selection

A two-variable analysis was performed for all blood cell count parameters considering their baseline values as continuous variables and the one-month differences as categorical. The PFS resulted negatively influenced by higher WBC value, with 0.6% higher probability of progression for every increase of 100 units/mm^3^ of the baseline values (HR = 1.006, p = 0.001) (Table 3). Higher values of WBC were also associated with a worse OS (HR = 1.009, p < 0.001) and lower response rate (OR = 1.017, p = 0.02) (Tables 4, 5). Regarding neutrophils, every increase of 100 units/mm^3^ at baseline was associated to a 1.1% higher probability of progression (HR 1.011, p < 0.001) (Table 3), 1.4% increase of risk of death (p < 0.001) and a 3% of worse response (p < 0.001) (Tables 4, 5).

Regarding the ratios, higher NLR values at baseline resulted associated with a worse PFS, OS and response. At baseline, each additional unit in the NLR value was associated to an increase of 17.6% of the risk of progression (p < 0.001), a 27.9% increase of the risk of death (p < 0.001) and a 32.9% increase in the risk of non-response (p < 0.001) (Tables 3, 4, 5). Higher values of PLR were also associated with worse OS (an increase of every unit was associated with a higher risk of death of 0.3%, p = 0.006). No significant correlation was found between PLR values and PFS or DCR (Tables 3, 4, 5).

**Fig. 1:**
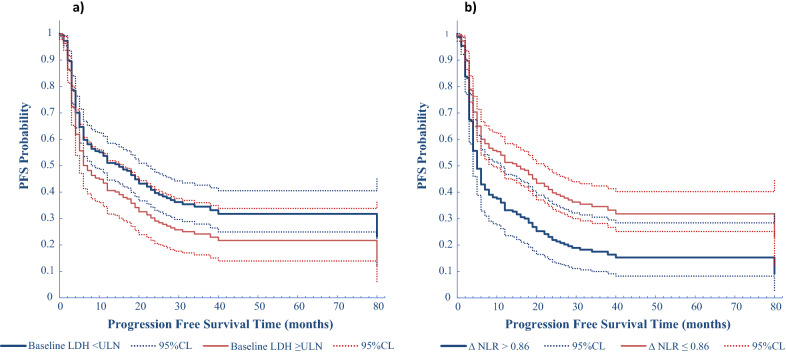
Direct adjusted survival probability for PFS from multivariable Cox model. Fig. 1**a** PFS probability by PFS time and LDH at baseline. **b** PFS probability by PFS time and ∆NLR

**Fig. 2:**
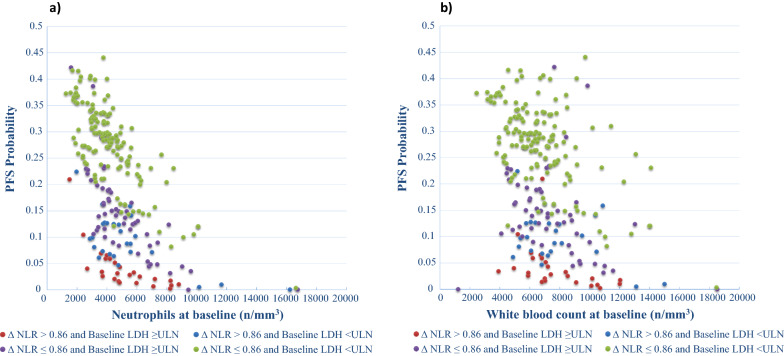
PFS probability at 80 months by Neutrophils, and WBC at baseline. All patients have been clustered in 4 groups, according to the combination of the values of the dichotomized variables basal LDH and ΔNLR, then associated with the values of the continuous variables baseline neutrophils (**a**) and baseline WBC count (**b**). Multivariable analysis results show a correlation between higher baseline neutrophils or WBC and progression. Risk of progression increases with increasing baseline neutrophils, more in those patients with risk factors (elevated LDH at baseline and elevated ΔNLR) than in patients with more favourable risk factors (ΔNLR < 0.86 and normal LDH at baseline). Risk of progression increases with increasing baseline WBC, more in those patients with risk factors (elevated LDH at baseline and elevated ΔNLR) than in patients with more favourable risk factors (ΔNLR < 0.86 and normal LDH at baseline)

In the multivariable analysis including both clinical and bio-humoral parameters, we found that the variables influencing PFS included LDH (p = 0.07) (Fig. 1a), neutrophils (p = 0.003) (Fig. 2a) and WBC (p = 0.069) (Fig. 2b), with neutrophils having the most significant impact (every increase of 100 units/mm^3^ was associated with a 2.2% increase in the risk of progression) (Table 3).

**Fig. 3:**
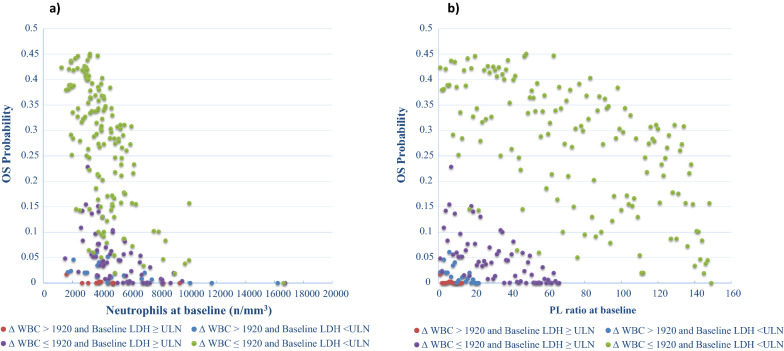
OS probability at 145 months by neutrophils and PLR at baseline. All patients have been clustered in 4 groups, according to the combination of the values of the dichotomized variables basal LDH and ΔWBC, then associated with the values of the continuous variables baseline neutrophils (**a**) and baseline PLR (**b**). Multivariable analysis results show a correlation between higher baseline WBC or neutrophils and lower OS. Risk of progression increases with increasing baseline neutrophils, more in those patients with risk factors (elevated LDH at baseline and elevated ΔWBC) than in patients with more favourable risk factors (ΔWBC < 1920/mm^3^ and normal LDH at baseline). **b** Risk of progression increases with increasing baseline PLR, more in those patients with risk factors (elevated LDH at baseline and elevated ΔNLR) than in patients with more favourable risk factors (ΔWBC < 1920/mm^3^ and normal LDH at baseline)

The OS was independently influenced by LDH (p < 0.001), neutrophils (every increase of 100 units/mm^3^ was associated with a 1.1% increase of the risk of death, p < 0.001) (Fig. 3a) and PLR (every increase of 1 unit was associated with a 0.3% higher risk of death, p = 0.022) (Fig. 3b and Table 4).

Finally, the DCR resulted mainly influenced by LDH (p = 0.032) and neutrophils (every increase of 100 units/mm^3^ was associated with a 2.2% risk of non-response, p = 0.004) (Table 5).

### Changes of blood parameters through the early phase of treatment

We then examined the changes in blood parameters after the first cycle of immunotherapy (3 weeks for pembrolizumab and ipilimumab; 4 weeks for nivolumab).

The difference between time 1 and time 0 (Δ) of WBC, neutrophils, lymphocytes, platelets and their ratios (NLR and PLR) were calculated and dichotomized using cut-off values assessed by ROC curve. Considering their baseline values as continuous variables and the one-month differences as categorical, in the two-variable regression model all investigated parameters resulted influencing the PFS: an increase in platelet values above the identified cut-off of 25,000/mm^3^ was associated with an increased risk of progression of 40% (HR 1.39, p = 0.039), as well as an increase of WBC greater than 1920 units/mm^3^ (HR = 2.063, p < 0.001), an increase of neutrophils greater than 310 units/mm^3^ (HR 1.429; p = 0.020) and a decrease in the value of lymphocytes more than 300 units/mm^3^ (HR = 1.910, p < 0.001). Patients with ΔNLR over the cut-off value of 0.86 had a worse PFS (HR = 1.975, p < 0.001), as well as patients with ΔPLR over 22.85 (HR = 1.693, p = 0.001) (Table 3).

All parameters were also found to influence significantly the OS: the increase above the cut-off values of WBC, neutrophils and platelets was associated with an increased risk of death (HR = 3.246, p < 0.001; HR = 1.880, p < 0.001, HR = 1.512, p = 0.022, respectively), as well as a decrease of lymphocytes over 300 units/mm^3^ (HR 2.319, p < 0.001) (Table 4). ΔNLR and ΔPLR remained also significant for OS: an increase of NLR and PLR above the cut-off is associated with a higher probability of death (HR = 2.908, p < 0.001 and HR = 2.019, p < 0.001, respectively) (Table 4). Moreover**,** all parameters resulted influencing the response: Δplatelets above the cut-off value was associated with a lower DCR (OR = 1.749, p = 0.043), as well as ΔWBC above the cut-off value (OR = 4.136, p < 0.001), Δneutrophils above the cut-off value (OR = 1.850, p < 0.021), and Δlymphocytes below the cut-off value (OR = 2.621, p < 0.002) (Table 5). Finally, a ΔNLR and ΔPLR above cut-off were associated with a lower response (OR = 2.779, p < 0.001, and OR = 2.022, p < 0.009, respectively) (Table 5).

**Fig. 4:**
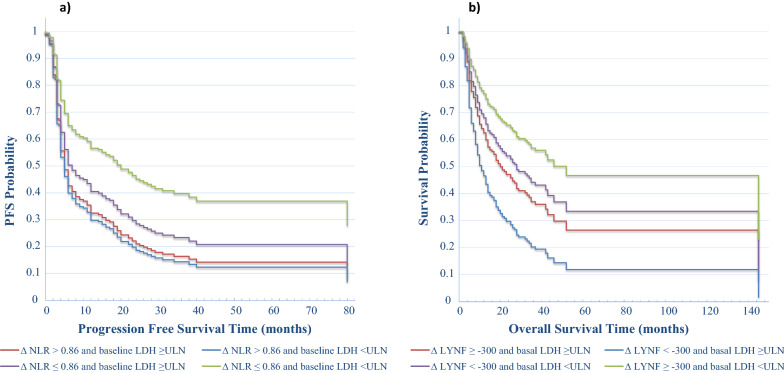
Direct adjusted survival probability for PFS (**a**) and OS (**b**) from multivariable Cox models. For different risk categories we used the combination of the basal values of LDH and the ΔNLR for PFS, and the combination of basal values of LDH and Δlymphocytes for OS. 3**a** For ∆NLR > 0.86 and basal LDH ≥ ULN the median PFS was 5 months; for ∆NLR > 0.86 and basal LDH < ULN the median PFS was 5 months; for ∆NLR ≤ 0.86 and basal LDH ≥ ULN the median PFS was 7 months; for ∆NLR ≤ 0.86 and basal LDH < ULN the median PFS was 20 months. 3b) For ∆lymphocytes < − 300 and basal LDH ≥ ULN the median OS was 11 months; for ∆lymphocytes ≥ − 300 and basal LDH ≥ ULN the median OS was 20 months; for ∆lymphocytes < − 300 and basal LDH < ULN the median OS was 28 months; for ∆lymphocytes ≥ − 300 and basal LDH < ULN the median OS was 52 months

In the multivariable model only ΔNLR resulted statistically significant for PFS (p = 0.004) with an increase above the cut-off value of 0.86 causing a 71% higher risk of progression (Fig. 1b, Table 3). Thus, we clustered our patients’ population in 4 groups according to basal LDH value and the ΔNLR. We found that LDH ≤ ULN and ΔNLR under the cut-off of 0.86 identified the patients’ population with the longest median PFS of 20 months with respect to that of patients with LDH > ULN and ΔNLR over the cut-off of 0.86 which had a median PFS of 5 months. Of interest, patients with normal LDH but with ΔNLR above the cut-off also have a worse median PFS of only 5 months (Fig. 4a).

**Fig. 5:**
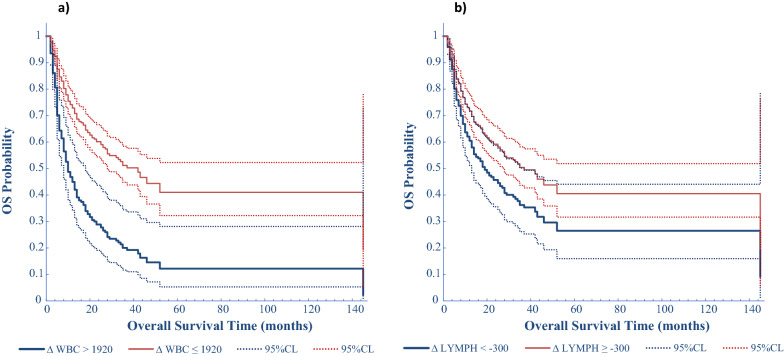
Direct adjusted survival probability for OS from multivariable Cox model. Figure 5**a** Survival probability by OS time and ∆WBC. **b** Survival probability by OS time and ∆LYMPH

Regarding the OS, the multivariate analysis showed a significant influence by ΔWBC, Δlymphocytes and PLR: an increase above the WBC cut-off value causes a 300% greater probability of death (HR = 2.918, p < 0.001) (Fig. 5a), and the decrease of lymphocytes greater than the cut-off of -300 units/mm^3^ is associated with a worse OS (HR = 1.627, p = 0.038) (Fig. 5b; Table 4). We also clustered the entire patient population in 4 categories according to basal LDH and Δlymphocytes. We found that LDH ≤ ULN and a higher lymphocytes count (Δ ≥ − 300/mm^3^) identified patients with the longest median OS of 52 months with respect of that of patients with LDH > ULN and a lower Δlymphocytes (< − 300/mm^3^) having a median OS of only 11 months (Fig. 4b).

Finally, when we combined ΔWBC and Δlymphocytes, we found a very long OS of 45 months for patients with ΔWBC lower and Δlymphocytes higher with respect to that of patients with ΔWBC higher and Δlymphocytes lower having an OS of only 7 months.

Regarding the DCR in the multivariable model, it resulted negatively associated with ΔWBC above the cut-off value (OR = 3.422, p = 0.003) (Table 5).

## Discussion

ICI therapy is the backbone of MM treatment, especially for patients without BRAF mutation for whom the anti-BRAF/anti-MEK target therapy is not suitable. The anti-PD-1 agents pembrolizumab and nivolumab give about 40% of response rate, with a PFS of 7–9 months and an OS of about 33–37 months [3, 4, 30].

In our retrospective cohort of patients, we reported an ORR of 41.9% with a DCR of 56.6%. The median PFS was 10 months, and the median OS was 29 months. This OS resulted a little shorter than that reported in large, controlled studies. The reasons could be that our patient population was quite different from that of controlled studies. First of all, we excluded patients harboring BRAF V600 mutations normally treated with targeted therapy as first line. Moreover, we had 10% of patients with brain metastases and 12% of patients with mucosal or uveal melanoma, that are notoriously poorly responsive to ICI therapy. Finally, 20.9% of our patients were treated with ipilimumab alone as first line, which gives a median OS of about 16–20 months [4.30]. When we recalculated PFS and OS by excluding patients with worse prognosis (brain metastases, mucosal melanoma, uveal melanoma), we reported a median PFS of 12 [7–19] months and a median OS of 42 [27–61] months, which are slightly better than those of pivotal studies.

Interestingly, increasing evidence are demonstrating that systemic and chronic inflammation mediated by different blood cell types and humoral factors induces an immunosuppressive status in the microenvironment that favors the growth and migration of cancer cells [31]. In this context, neutrophils and platelets play a relevant role [6–10, 32]. Neutrophils have been reported to support the development of metastases through multiple mechanisms such as the release of proteases that degrade antitumoral factors, the production of leukotrienes that propagate metastasis-initiating cells, the inhibition of antitumor T cell responses [11, 33]. In contrast, tumor growth is inhibited when migration of neutrophils to tumoral areas or granulocyte colony stimulating factors are blocked [34]. Neutrophils and NLR have been reported as prognostic markers in several types of neoplasms [35] as renal cell carcinoma [32], non-small cell lung cancer [36], breast cancer [37], colorectal cancer [38].

Also, platelets and PLR have been found to be independent prognostic factors for numerous solid tumors [39] including non-small cell lung cancer [36], hepatocellular carcinoma [12], renal cell carcinoma [32], breast cancer [13], colorectal cancer [40] and melanoma [24].

Recent findings demonstrated that also the response to ICI is influenced by the inflammation status. Higher levels of neutrophils have been found associated with less benefit from ICI treatment in different types of cancer [14–16, 19–23]. Moreover, Zhou and colleagues used a whole-blood multicolor flow cytometry in patients with metastatic cancers to identify a liquid immune profile signature based on five cell subtypes significantly associated with a better OS: CD14^high^ monocytes, CD8 + /PD-1^+^ T cells, plasmacytoid dendritic cells, neutrophils, and CD3^+^/CD56^+^/CD16^+^ natural killer T cells. Of note, in the time point after the first administration of ICI, only natural killer T cells and neutrophils still maintained their predictive value for OS and PFS, underlining the relevant role played by neutrophils in the responsiveness to ICI immunotherapy [41].

NLR and PLR were also reported as strong prognostic markers in patients with non-small cell lung cancer treated with nivolumab [42]. Furthermore, a pan-immune-inflammation value (PIV) involving the neutrophil, platelet and monocyte count was significantly correlated with worse clinical outcomes in 163 microsatellite instability-high metastatic colorectal cancer patients treated with ICI [38].

In MM, it has been reported that basal neutrophils and NLR are prognostic in patients receiving ipilimumab [43] or anti-PD-1 [21, 23]. These data have been recently confirmed in a pooled from MM patients accrued in three large phase II/III randomized ICI trials, one of which included a chemotherapy arm [44]. Higher values of NLR, interleukin-6 and C reactive protein were associated with a shorter survival in patients receiving ICI or chemotherapy [44]. Finally, in a retrospective study including 72 patients with MM and 84 with non-small-cell lung cancer treated with PD-1 inhibitors, Kartolo and colleagues reported that a baseline value of NLR ≥ 5 and PLR ≥ 200 were independently associated with worse ICI efficacy regardless of cancer type. In addition, patients with both high NLR and PLR had the worst treatment outcome when compared to patients with only one elevated biomarker or none [15].

In our large population of 272 patients, in addition to confirming that baseline higher LDH value and higher number of metastatic sites closely correlated with poorer clinical outcomes, we found that higher values of neutrophils and NLR had a negative impact on PFS, OS and response rate. Also, higher values of PLR were associated with a worse OS but not on PFS or DCR. At the multivariable analysis including both clinical and bio-humoral parameters, we found that LDH (p = 0.07)**,** WBC count (p = 0.069) and neutrophils (p = 0.003) strongly influenced PFS with neutrophils having the greatest weight on this clinical outcome. Moreover, the OS was independently influenced by LDH (p < 0.001), neutrophils (p < 0.001) and PLR (p = 0.022). Finally, the DCR resulted mainly influenced by basal LDH (p = 0.03) and neutrophils (p = 0.004).

Of interest, some recent reports underlined that early changes of these simple hematological parameters during ICI therapy increase the capacity to distinguish responder from non-responder patients. However, very few data are currently available on this topic in particular regarding early variations of platelets and PLR.

In 197 patients with advanced melanoma treated with ipilimumab, Cassidy and colleagues reported that an increase of 30% in the NLR during treatment was strongly associated with worse OS. Interestingly, this change was not prognostic in the 65 patients treated with BRAF inhibitors leading authors to conclude that NLR may have a uniquely predictive value in patients treated with ICI [26]. Another study in patients with renal cell carcinoma treated with ICI demonstrated that a ≥ 25% increase in NLR resulted strongly associated with a worse PFS and OS [27]. More recently, in 224 patients with advanced melanoma treated with PD-1 inhibitor monotherapy, while confirming the independent predictive value of baseline NLR on survival and its correlation with performance status and number of metastatic sites, Bartlett and colleagues also found that an increase in NLR ≥ 30% during the first 2 cycles of treatment was associated with a significantly worse OS and with a trend towards shorter time to treatment failure. Interestingly, combining baseline NLR ≥ 5 and a NLR increase ≥ 30%, the Authors identified a small cohort of patients with a very poor OS [16]. In a small group of 16 MM patients treated with anti-PD1, Ohashi and colleagues found that responders presented an increase of lymphocytes and eosinophils and a decrease of neutrophils within the first 6 weeks of treatment [25]. Finally, Corti and colleagues reported that an early increase of pan-immune-inflammation value (PIV) involving the neutrophil, platelet and monocyte count, resulted an independent predictor of clinical benefit in metastatic colorectal cancer patients treated with immune checkpoint inhibitors [38].

Nevertheless, although confirming the predictive role of 6-week NLR and PLR, other Authors were not able to establish an optimal cut-off for ΔNLR and ΔPLR in prognosticating treatment efficacy, probably due to the fact that the majority of patients maintained high values of NLR and PLR at 6-week of ICI therapy [15].

In our paper, we showed that variations between time 1 and time 0 (Δ) of platelets, WBC, neutrophils, and lymphocytes strongly influenced the PFS in a two-variable modelMoreover, patients with ΔNLR superior to the cut-off had a worse PFS (HR = 1.975, p < 0.001), as well as patients with ΔPLR over the cut-off (HR = 1.693, p = 0.001). In our experience, all these parameters have also significantly affected the OS and response.

Nevertheless, in the multivariable model only ΔNLR resulted statistically significant on PFS (p = 0.004) with an increase above the cut-off causing a 71% higher risk of progression. Interestingly, when we clustered our patient population in 4 groups according to basal LDH value and the ΔNLR, we found that LDH ≤ ULN and ΔNLR under the cut-off identified a sub-group of patients with the longest median PFS of 20 months versus only 5 months of patients with both these parameters elevated. Of interest, patients with normal LDH but with ΔNLR above the cut-off also had a median PFS of about 5 months showing that ΔNLR had the strongest impact on PFS outcome.

The OS, in multivariable analysis, resulted influenced by WBC, lymphocytes and PLR variations with ΔWBC and Δlymphocytes having a statistically significant: the increase above the WBC cut-off causes a 92% greater probability of death (p < 0.001) and the decrease of lymphocytes greater than the cut-off is associated with a worse OS (HR = 1.627, p = 0.038).

When we clustered the entire patient population in 4 categories according to basal LDH and Δlymphocytes, we found that LDH ≤ ULN and a higher Δlymphocytes identified patients with the longest median OS of 50 months versus only 10 months of patients with LDH > ULN and a lower Δlymphocytes. Also, combining ΔWBC and Δlymphocytes, we found that ΔWBC lower and Δlymphocytes higher identified patients with a longer OS of 45 months versus 7 months of patients with ΔWBC higher and Δlymphocytes lower.

Finally, in the multivariable model, the DCR was associated with changes in WBC and lymphocytes, but only the increase of WBC above the cut-off value resulted significantly associated with a poorer DCR (OR = 3.422, p = 0.003).

Multiple NLR and PLR cut-offs were suggested by previous studies for different cancer types including melanoma [15, 16, 21, 45]. For clinical practice purposes, it would be convenient for clinicians to have unifying NLR and PLR cut-off values regardless of cancer types.

We used a two-variables regression model for all haematological parameters considering their baseline values as continuous variables and the one-month differences as categorical. Then, the difference between time 1 and time 0 of WBC, neutrophils, lymphocytes, platelets and their ratios (NLR and PLR) were calculated and dichotomized using cut-off values assessed by ROC curve. For the analysis of the one-month differences in haematological parameters, two-variable regression was used to take into account for possible confounding influences of their baseline values.

The novelty and strength of our work compared to the others previously published include: homogeneity and large size of our population with all patient being BRAF wild type and treated with checkpoint inhibitors as first line; evaluation of all parameters studied both at baseline and after one course of ICI therapy; addition of the role of platelets and PLR, hitherto little studied, as predictors of response to ICI. All these simple and cost-effective hematological parameters, both at basal and as early variations, resulted to predict the efficacy of treatment in a large population of MM.

Due to the facility to obtain these parameters through routine procedures, they could be used as potential biomarkers in decisions about candidacy allowing clinicians to choose patients most likely to have a clinical benefit to ICI therapy, and for stratification in clinical trials. Moreover, their early variations during treatment may serve as a simple monitoring tool to evaluated the continuation of therapy in the case of uncertain clinical outcomes.

There were some limitations in our study that require attention. First of all, the retrospective nature of our study. Moreover, the heterogeneity of our patient’s population (10% with brain metastases, 12% with mucosal or uveal melanoma, 20.9% treated with ipilimumab alone) and the exclusion of BRAF V600 mutated patients. Lastly, in the absence of standardized values that define cut-off for these hematological parameters and for their ratios, we also used arbitrary cut-offs obtained from the ROC curves of OS. To overcome these limitations, further evaluations in larger multicenter prospective studies are warranted.

## Data Availability

The datasets used and/or analysed during the current study are available from the corresponding author on reasonable request.
